# Variants of the Aggression-Related *RBFOX1* Gene in a Population Representative Birth Cohort Study: Aggressiveness, Personality, and Alcohol Use Disorder

**DOI:** 10.3389/fpsyt.2020.501847

**Published:** 2020-11-24

**Authors:** Mariliis Vaht, Kariina Laas, Noèlia Fernàndez-Castillo, Triin Kurrikoff, Margus Kanarik, Stephen V. Faraone, Liina-Mai Tooding, Toomas Veidebaum, Barbara Franke, Andreas Reif, Bru Cormand, Jaanus Harro

**Affiliations:** ^1^Division of Neuropsychopharmacology, Department of Psychology, Estonian Center of Behavioral and Health Sciences, University of Tartu, Tartu, Estonia; ^2^Departament de Genètica, Microbiologia i Estadística, Facultat de Biologia, Universitat de Barcelona, Barcelona, Spain; ^3^Centro de Investigación Biomédica en Red de Enfermedades Raras (CIBERER), Instituto de Salud Carlos III, Madrid, Spain; ^4^Institut de Biomedicina de la Universitat de Barcelona (IBUB), Barcelona, Spain; ^5^Institut de Recerca Sant Joan de Déu (IR-SJD), Esplugues de Llobregat, Spain; ^6^Institute of Social Studies, University of Tartu, Tartu, Estonia; ^7^Departments of Psychiatry and of Neuroscience and Physiology, The State University of New York Upstate Medical University, Syracuse, NY, United States; ^8^National Institute for Health Development, Estonian Center of Behavioral and Health Sciences, Tallinn, Estonia; ^9^Department of Human Genetics, Radboud University Medical Center, Donders Institute for Brain, Cognition and Behavior, Nijmegen, Netherlands; ^10^Department of Psychiatry, Psychosomatic Medicine and Psychotherapy, University Hospital Frankfurt Goethe University, Frankfurt am Main, Germany

**Keywords:** *RBFOX1*, A2BP1, aggressiveness, neuroticism, extraversion, alcohol use disorder, gender

## Abstract

**Background:** Recently, *RBFOX1*, a gene encoding an RNA binding protein, has consistently been associated with aggressive and antisocial behavior. Several loci in the gene have been nominally associated with aggression in genome-wide association studies, the risk alleles being more frequent in the general population. We have hence examined the association of four *RBFOX1* single nucleotide polymorphisms, previously found related to aggressive traits, with aggressiveness, personality, and alcohol use disorder in birth cohort representative samples.

**Methods:** We used both birth cohorts of the Estonian Children Personality Behavior and Health Study (ECPBHS; original *n* = 1,238). Aggressiveness was assessed using the Buss–Perry Aggression Questionnaire and the Lifetime History of Aggressiveness structured interview at age 25 (younger cohort) or 33 (older cohort). Big Five personality at age 25 was measured with self-reports and the lifetime occurrence of alcohol use disorder assessed with the MINI interview. *RBFOX1* polymorphisms rs809682, rs8062784, rs12921846, and rs6500744 were genotyped in all participants. Given the restricted size of the sample, correction for multiple comparisons was not applied.

**Results:** Aggressiveness was not significantly associated with the *RBFOX1* genotype. *RBFOX1* rs8062784 was associated with neuroticism and rs809682 with extraversion. Two out of four analyzed *RBFOX1* variants, rs8062784 and rs12921846, were associated with the occurrence of alcohol use disorder.

**Conclusions:** In the birth cohort representative sample of the ECPBHS, no association of *RBFOX1* with aggressiveness was found, but *RBFOX1* variants affected basic personality traits and the prevalence of alcohol use disorder. Future studies on *RBFOX1* should consider the moderating role of personality and alcohol use patterns in aggressiveness.

## Introduction

Globally, more than 1.3 million people worldwide die each year owing to aggressive behavior and violence (either self-directed, interpersonal, and collective), accounting for 2.5% of mortality (1). Nevertheless, from the evolutionary perspective, aggression can be described as adaptive. Aggression belongs to the behavioral repertoire of most species. Humans are a rather highly aggressive species compared with other animals. This may be related to the high benefit-to-cost ratio for intraspecific aggression (2). Aggression in mammals, including humans, has a high heritability (3, 4). Struggle for resources (e.g., territory, suitable mates, food) must have substantially contributed to the shaping of aggression by favoring gene variants that promote agonistic behavior. However, when humans express their inherent aggressiveness in an unfitting context, this may lead to social maladjustment and crime (5).

Variance in many genes has been associated with aggressiveness, with very small effects of each in the large population studies by GWAS (6, 7). Recently, a novel candidate gene, *RBFOX1*, has been linked to aggressive behavior by convergent evidence from GWAS, epigenetic analyses, and neuroimaging genetics, gene expression, and animal models (8). *RBFOX1* (RNA binding protein, Fox-1 homolog 1; also known as ataxin 2-binding protein 1, A2BP1, or hexaribonucleotide-binding protein 1, HRNBP1) encodes for the Fox-1 protein and regulates alternative splicing that controls gene expression and, in turn, coordinates neuronal brain activity (9, 10).

RNA binding proteins (RBPs) are key components in RNA metabolism (11) by influencing the structure and interactions of the RNAs and playing critical roles in their biogenesis, stability, function, transport, and cellular localization (12). Each RBP interacts with RNA in a unique sequence- or structure-specific manner. Alternative splicing is one of the central mechanisms regulating eukaryotic gene expression (13). RBPs coordinate elaborate networks of RNA–protein and protein–protein interactions that control RNA metabolism. Hence, alterations in their RNA-binding function could impact many genes and pathways, leading to complex, multifaceted phenotypes (11, 14). Mutations in RBPs cause and/or contribute to many human neurodevelopmental and neurologic disorders (11, 15, 16). Abnormalities in the Fox-1 encoding gene, *RBFOX1*, are associated with neurodevelopmental disorders (17). Variations in the *RBFOX1* have been associated with anxiety (9), attention deficit/hyperactivity disorder (18), schizophrenia (19, 20), autism spectrum disorders (17), intellectual disability with epilepsy (21), and gray matter loss in Alzheimer's disease patients (22). Conclusively, potential effects of the *RBFOX1* genotype are multiple and likely variable by sample specifics owing to environmental interactions.

Several variants of *RBFOX1* have been associated with aspects of aggressiveness in a number of GWASs (23). The C allele of rs6500744, located within the first intron, was associated with conduct disorder symptoms in the interaction with mothers' warmth (24). Another SNP (rs8062784) in intron 1 of *RBFOX1* was associated with anger in a GWAS assessing hostility (25), and a variant located in intron 3 of the gene (rs12921846) was associated with conduct disorder in a sample of ADHD trios (26). A meta-analysis of nine population-based GWASs including around 19,000 children provided evidence for the contribution of *RBFOX1* to children's aggressive behavior (7). Four SNPs in the *RBFOX1* gene (rs809682, rs12922093, rs12373031, and rs10521042, all located in the intron regions) showed suggestive associations. Of these, the rs809682 polymorphism was demonstrated to have the lowest association *p*-value, with the major T allele conferring the risk. Statistically significant association was found for rs809682 when comparing aggressive prisoners to controls and non-aggressive prisoners (23). It should be noticed though that the direction of the effect was not as in the original study.

We selected the SNPs showing the lowest association *p*-value from every single reported GWAS [rs6500744, rs8062784, rs809682, and rs12921846; see (23)] and assessed their association with aggressiveness and basic personality traits in a birth cohort representative sample of young adults. Given that antisocial behavior is the main predictor of alcohol (mis)use and the other way around [e.g., (27)], we also examined the occurrence of alcohol use disorder.

## Materials and Methods

### Study Population

The analysis was carried out on the Estonian Children Personality Behavior and Health Study (ECPBHS) sample, the original Estonian sample of the European Youth Heart Study (1998/99) that was subsequently incorporated into the longitudinal ECPBHS. All the subjects are of European descent. The principles of formation of the original sample and procedure of first data collection have been described in detail elsewhere (28). In brief, this is a representative birth cohort sample of the Tartu city and county with a school as the sampling unit. All schools of Tartu County, Estonia, that agreed to participate (54 of the total of 56) were included into the sampling using the probability proportional to the number of students of the respective age groups in the school, and 25 schools were selected. All children from grades 3 (younger birth cohort) and 9 (older birth cohort) were invited to participate. ECPBHS is population representative, while 79.1% of subjects of the randomized regional sample participated in the original sampling. Details on follow-ups have been described elsewhere [e.g., (29)], and the number of participants in the presented analyses is given below. The study was approved by the Ethics Review Committee on Human Research of the University of Tartu. Written informed consent was obtained from all participants and, in case of minors, also from their parents.

### Measures

#### Aggressive Behavior

During the last data collection waves (at age 25 years in the younger cohort in 2014/2015 and at age 33 in the older cohort in 2016/2017), aggressive behavior was self-reported using the Buss-Perry Aggression Questionnaire (30). The 29-item self-report Buss-Perry Aggression Questionnaire (31) assesses four aspects of aggressive behavior: Physical aggression, Verbal aggression, Anger, and Hostility. Participants (*n* = 436 in the younger and *n* = 499 in the older cohort) rated each statement on a 5-point Likert Scale (uncharacteristic = 1, characteristic = 5). During the same data collection waves, the Life History of Aggression interview [LHA; (32)] was carried out by experienced clinical psychologists in order to assess dimensions of aggression (*n* = 427 in the younger and *n* = 495 in the older cohort). Items were scored only for the history of actual behavior (33). LHA has three subscales: Aggression (temper tantrums, physical fighting, verbal fighting, assaults on other people, and assaults on property); Consequences/Antisocial Behavior (school disciplinary problems, problems with supervisors at work, antisocial behavior not resulting in police involvement, and antisocial behavior involving the police); and Self-Directed Aggression (assaults on self and suicide attempts). Each item was rated on a 5-point scale, ranging from 0 = “no events” to 5 = “more events than can be counted.”

#### Personality

Personality traits of the five-factor model (34) were measured by self-reports at age 25 (*n* = 856) with EE.PIP-NEO (35), which is a semantically simplified 240-item version of the International Personality Item Pool (IPIP), which emulates the NEO-PI-R.

#### Alcohol Use Disorders

Assessment of lifetime occurrence of alcohol use disorders was based on DSM-IV and was carried out in both cohorts (*n* = 931) at age 25 by experienced clinical psychologists using the Mini-International Neuropsychiatric Interview [M.I.N.I.5.0.0; (36); Estonian version; (37)].

### Genotyping

Genomic DNA was extracted from whole blood samples using Qiagen QIAamp® DNA Blood Midi Kit. The quantitative real-time polymerase chain reaction (qRT-PCR) for genotyping the four SNP polymorphisms was performed using TaqMan Pre-Designed SNP Genotyping Assays (Applied Biosystems; Foster City, CA, United States) containing primers and fluorescent probes. For rs809682, the Assay C___8926788_10 was utilized; for rs8062784, rs12921846, and rs6500744 polymorphisms, the Assays C__29081048_20, C__32104163_10, and C___3008571_10 were used, respectively. Genotyping reactions were performed in a total volume of 10 ml with ~25 ng of template DNA. QRT-PCR reaction components and final concentrations were as follows: 1:5 5 x HOT FIREPol® Probe qPCR Mix Plus (ROX) (Solis BioDyne) and 1:20 80 × TaqMan Primers Probe.

Context sequences [VIC/FAM] were as follows: rs809682–TAACAAACTACAGCCTAATTTAGTA[A/T]GTGAACTAAGTGAAAGCCTAACTTG, rs8062784–TTCTGTGAACCAACACTTTCTTTTC[A/T]TTGGGTTTGATACAGTGGCATCAAT,rs12921846–ATCTTGGAAAGCATTTGTTATTTCA[A/T]ACTCTTCAAATCTGCAAGTCTTACA, rs6500744–GCTTACCATTTATTTTATTTTCAGG[C/T]GGTTGTATTCATTATAATGCCATTA.

Reactions were performed on the Applied Biosystems ViiA™ 7 Real-Time PCR System. The amplification procedure consisted of an initial denaturation step at 95°C for 12 min and 40 cycles of 95°C for 15 s and 60°C for 1 min. Positive and negative controls were added to each reaction plate. No inconsistencies occurred. Genotyping was performed blind to all phenotypic data. Allele frequencies agreed with the National Center for Biotechnology Information database and published reports. Genotype frequencies were in Hardy-Weinberg equilibrium and are shown in Table 1.

**Table 1:** *RBFOX1* genotype frequencies in the ECPBHS sample.

**SNP**	**rs6500744**	**rs8062784**	**rs809682**	**rs12921846**
genotypes	CC/CT/TT	AA/AT/TT	TT/AT/AA	AA/AT/TT
MAF^#^ in the whole sample	MAF(T) = 0.37	MAF(T) = 0.06	MAF(A) = 0.26	MAF(T) = 0.21
Older cohort (*n* = 653)	262/300/91	580/70/3	342/268/43	399/229/25
Males (*n* = 290)	117/136/37	261/27/2	148/127/15	177/102/11
Females (*n* = 3 63)	145/164/54	319/43/1	194/141/28	222/127/14
Younger cohort (*n* = 580)	227/272/81	513/66/1	324/219/37	369/180/31
Males (*n* = 277)	115/118/44	239/37/1	168/95/14	173/92/12
Females (*n* = 303)	112/154/37	274/29/0	156/124/23	196/88/19

# *MAF, minor allele frequency*.

### Statistical Analysis

Birth cohorts were pooled for analysis. Categorical variable (genotype) relations to continuous variables were explored with analysis of variance (ANOVA) and presented as *F*-statistic, raw *p*-value and eta-squared (η^2^) as a measure of effect size. Fisher's least significance difference method (LSD) was used in all *post hoc* comparisons. Contrasts were calculated for significant model effects. Chi-square tests were conducted to assess the distribution of Alcohol Use Disorder by genotype, and by genotype and gender, and presented as χ^2^-statistic and raw *p*-value. For fitting of the path model, we used the AMOS package of structural equation modeling (SEM) by the MCMC (Markov chain Monte Carlo) method. Genotype was entered into the SEM model as a dichotomous variable: A/A homozygotes vs. T-allele carriers. All *p*-values are reported as two-tailed, and results are considered significant at the conventional *p* < 0.05 level; correction for multiple testing is not applied. Statistical analyses were performed using IBM SPSS Statistics, Version 25.

## Results

### The Selected *RBFOX1* Polymorphisms and Aggressive Behavior

Aggressiveness assessed either using the self-report Buss-Perry Aggression Questionnaire (Table 2) or the Lifetime History of Aggression interview (Table 3) was not associated with any of the *RBFOX1* polymorphisms. This was the case for total scores as well as subscales. No genotype by gender effect was found either.

**Table 2:** *RBFOX1* genotypes and the Buss-Perry Aggression Questionnaire at age 25.

	**Genotype main effects**		**Genotype by gender effects**		
	**Main statistics**	**Scores**	**Main statistics**	**Scores for males**	**Scores for females**
**rs809682**					
Physical aggression	*F*_(2, 923)_ = 0.07, *p* = 0.937, η^2^ <0.001	AA = 16.53 ± 0.77 AT = 16.53 ± 0.31 TT = 16.67 ± 0.27	*F*_(2, 920)_ = 0.94, *p* = 0.391, η^2^ = 0.002	AA = 19.52 ± 1.27 AT = 19.04 ± 0.50 TT = 18.46 ± 0.41	AA = 14.77 ± 0.88 AT = 14.83 ± 0.37 TT = 15.20 ± 0.33
Verbal Aggression	*F*_(2, 923)_ = 1.23, *p* = 0.292, η^2^ = 0.003	AA = 14.07 ± 0.49 AT = 13.61 ± 0.20 TT = 13.34 ± 0.18	*F*_(2, 920)_ = 0.01, *p* = 0.986, η^2^ <0.001	AA = 14.87 ± 0.78 AT = 14.38 ± 0.31 TT = 14.10 ± 0.25	AA = 13.60 ± 0.63 AT = 13.09 ± 0.26 TT = 12.72 ± 0.24
Anger	*F*_(2, 923)_ = 0.58, *p* = 0.558, η^2^ = 0.001	AA = 15.23 ± 0.66 AT = 15.75 ± 0.27 TT = 15.40 ± 0.23	*F*_(2, 920)_ = 0.48, *p* = 0.619, η^2^ = 0.001	AA = 15.35 ± 1.07 AT = 15.19 ± 0.42 TT = 14.74 ± 0.34	AA = 15.17 ± 0.83 AT = 16.17 ± 0.35 TT = 15.95 ± 0.32
Hostility	*F*_(2, 923)_ = 0.98, *p* = 0.375, η^2^ = 0.002	AA = 18.63 ± 0.71 AT = 17.67 ± 0.29 TT = 17.58 ± 0.25	*F*_(2, 920)_ = 0.71, *p* = 0.494, η^2^ = 0.002	AA = 19.83 ± 1.08 AT = 17.72 ± 0.42 TT = 17.88 ± 0.35	AA = 17.92 ± 0.93 AT = 17.63 ± 0.39 TT = 17.33 ± 0.35
BP total	*F*_(2, 923)_ = 0.30, *p* = 0.742, η^2^ = 0.001	AA = 64.46 ± 2.04 AT = 63.55 ± 0.83 TT = 62.99 ± 0.72	*F*_(2, 920)_ = 0.44, *p* = 0.644, η^2^ = 0.001	AA = 69.57 ± 3.31 AT = 66.33 ± 1.30 TT = 65.18 ± 1.07	AA = 61.45 ± 2.54 AT = 61.67 ± 1.07 TT = 61.19 ± 0.97
**rs8062784**					
Physical aggression	*F*_(2, 924)_ = 0.22, *p* = 0.642, η^2^ <0.001	AA = 16.57 ± 0.21 T-all = 16.86 ± 0.59	*F*_(2, 922)_ = 0.04, *p* = 0.852, η^2^ <0.001	AA = 18.74 ± 0.34 T-all = 18.76 ± 0.87	AA = 14.99 ± 0.25 T-all = 15.23 ± 0.72
Verbal aggression	*F*_(2, 924)_ = 0.05, *p* = 0.822, η^2^ <0.001	AA = 13.49 ± 0.14 T-all = 13.58 ± 0.38	*F*_(2, 922)_ = 0.37, *p* = 0.546, η^2^ <0.001	AA = 14.21 ± 0.20 T-all = 14.51 ± 0.54	AA = 12.96 ± 0.18 T-all = 12.77 ± 0.52
Anger	*F*_(2, 924)_ = 0.13, *p* = 0.721, η^2^ <0.001	AA = 15.51 ± 0.18 T-all = 15.70 ± 0.50	*F*_(2, 922)_ = 0.81, *p* = 0.370, η^2^ = 0.001	AA = 14.98 ± 0.28 T-all = 14.69 ± 0.73	AA = 15.89 ± 0.24 T-all = 16.56 ± 0.69
Hostility	*F*_(2, 924)_ = 0.21, *p* = 0.646, η^2^ <0.001	AA = 17.65 ± 0.19 T-all = 17.92 ± 0.54	*F*_(2, 922)_ = 3.62, *p* = 0.057, η^2^ = 0.004	AA = 18.05 ± 0.28 T-all = 17.11 ± 0.74	AA = 17.36 ± 0.27 T-all = 18.61 ± 0.77
BP total	*F*_(2, 924)_ = 0.26, *p* = 0.614, η^2^ <0.001	AA = 63.22 ± 0.56 T-all = 64.05 ± 1.56	*F*_(2, 922)_ = 0.77, *p* = 0.380, η^2^ = 0.001	AA = 65.99 ± 0.86 T-all = 65.08 ± 2.27	AA = 61.20 ± 0.73 T-all = 63.18 ± 2.10
**rs12921846**					
Physical aggression	*F*_(2, 923)_ = 0.34, *p* = 0.715, η^2^ = 0.001	AA = 16.56 ± 0.25 AT = 16.74 ± 0.34 TT = 15.92 ± 0.97	*F*_(2, 920)_ = 0.34, *p* = 0.712, η^2^ = 0.001	AA = 18.70 ± 0.39 AT = 18.77 ± 0.52 TT = 19.29 ± 1.63	AA = 15.0 ± 0.30 AT = 15.19 ± 0.41 TT = 14.04 ± 1.09
Verbal aggression	*F*_(2, 923)_ = 0.75, *p* = 0.474, η^2^ = 0.002	AA = 13.41 ± 0.16 AT = 13.57 ± 0.22 TT = 14.15 ± 0.62	*F*_(2, 920)_ = 0.78, *p* = 0.459, η^2^ = 0.002	AA = 14.31 ± 0.24 AT = 14.07 ± 0.32 TT = 15.0 ± 1.0	AA = 12.74 ± 0.22 AT = 13.19 ± 0.29 TT = 13.68 ± 0.78
Anger	*F*_(2, 923)_ = 0.37, *p* = 0.688, η^2^ = 0.001	AA = 15.42 ± 0.22 AT = 15.68 ± 0.29 TT = 15.92 ± 0.83	*F*_(2, 920)_ = 0.82, *p* = 0.440, η^2^ = 0.002	AA = 14.72 ± 0.33 AT = 15.18 ± 0.44 TT = 16.57 ± 1.37	AA = 15.94 ± 0.29 AT = 16.07 ± 0.39 TT = 15.56 ± 1.04
Hostility	*F*_(2, 923)_ = 1.14, *p* = 0.322, η^2^ = 0.002	AA = 17.47 ± 0.23 AT = 18.04 ± 0.31 TT = 18.00 ± 0.89	*F*_(2, 920)_ = 0.22, *p* = 0.800, η^2^ <0.001	AA = 17.83 ± 0.33 AT = 18.10 ± 0.45 TT = 18.14 ± 1.39	AA = 17.20 ± 0.32 AT = 17.99 ± 0.43 TT = 17.92 ± 1.16
BP total	*F*_(2, 923)_ = 0.56, *p* = 0.571, η^2^ = 0.001	AA = 62.88 ± 0.67 AT = 64.03 ± 0.90 TT = 64.0 ± 2.57	*F*_(2, 920)_ = 0.30, *p* = 0.739, η^2^ = 0.001	AA = 65.56 ± 1.02 AT = 66.12 ± 1.37 TT = 69.0 ± 4.25	AA = 60.88 ± 0.88 AT = 62.43 ± 1.19 TT = 61.20 ± 3.17
**rs6500744**					
Physical aggression	*F*_(2, 923)_ = 0.77, *p* = 0.462, η^2^ = 0.002	CC = 16.37 ± 0.31 CT = 16.87 ± 0.29 TT = 16.42 ± 0.54	*F*_(2, 920)_ = 0.18, *p* = 0.837, η^2^ <0.001	CC = 18.60 ± 0.48 CT = 19.03 ± 0.46 TT = 18.24 ± 0.82	CC = 14.65 ± 0.38 CT = 15.34 ± 0.35 TT = 14.99 ± 0.65
Verbal aggression	*F*_(2, 923)_ = 0.61, *p* = 0.543, η^2^ = 0.001	CC = 13.63 ± 0.20 CT = 13.47 ± 0.19 TT = 13.19 ± 0.35	*F*_(2, 920)_ = 1.07, *p* = 0.342, η^2^ = 0.002	CC = 14.33 ± 0.29 CT = 14.42 ± 0.28 TT = 13.47 ± 0.50	CC = 13.10 ± 0.27 CT = 12.79 ± 0.25 TT = 12.97 ± 0.47
Anger	*F*_(2, 923)_ = 2.27, *p* = 0.104, η^2^ = 0.005	CC = 15.59 ± 0.27 CT = 15.74 ± 0.25 TT = 14.63 ± 0.46	*F*_(2, 920)_ = 0.17, *p* = 0.843, η^2^ <0.001	CC = 14.89 ± 0.40 CT = 15.26 ± 0.38 TT = 14.09 ± 0.69	CC = 16.13 ± 0.36 CT = 16.08 ± 0.33 TT = 15.05 ± 0.62
Hostility	*F*_(2, 923)_ = 2.09, *p* = 0.124, η^2^ = 0.005	CC = 17.78 ± 0.29 CT = 17.87 ± 0.27 TT = 16.75 ± 0.50	*F*_(2, 920)_ = 0.31, *p* = 0.737, η^2^ = 0.001	CC = 17.92 ± 0.41 CT = 18.12 ± 0.39 TT = 17.39 ± 0.70	CC = 17.67 ± 0.40 CT = 17.70 ± 0.37 TT = 16.24 ± 0.69
BP total	*F*_(2, 923)_ = 1.66, *p* = 0.191, η^2^ = 0.004	CC = 63.37 ± 0.83 CT = 63.95 ± 0.78 TT = 60.99 ± 1.43	*F*_(2, 920)_ = 0.08, *p* = 0.927, η^2^ <0.001	CC = 65.74 ± 1.24 CT = 66.84 ± 1.19 TT = 63.20 ± 2.14	CC = 61.55 ± 1.09 CT = 61.90 ± 1.01 TT = 59.26 ± 1.90

**Table 3:** *RBFOX1* genotypes and the life history of aggression interview at age 25.

	**Genotype main effects**		**Genotype by gender effects**		
	**Main statistics**	**Scores**	**Main statistics**	**Scores for males**	**Scores for females**
**rs809682**					
Aggression	*F*_(2, 919)_ = 0.14, *p* = 0.872, η^2^ <0.001	AA = 6.27 ± 0.55 AT = 5.97 ± 0.22 TT = 6.0 ± 0.20	*F*_(2, 916)_ = 0.79, *p* = 0.453, η^2^ = 0.002	AA = 7.23 ± 0.98 AT = 7.34 ± 0.37 TT = 6.90 ± 0.31	AA = 5.75 ± 0.62 AT = 4.99 ± 0.26 TT = 5.24 ± 0.24
Antisocial behavior	*F*_(2, 919)_ = 1.43, *p* = 0.240, η^2^ = 0.003	AA = 1.40 ± 0.36 AT = 2.04 ± 0.15 TT = 1.88 ± 0.13	*F*_(2, 916)_ = 1.67, *p* = 0.188, η^2^ = 0.004	AA = 2.86 ± 0.73 AT = 3.89 ± 0.27 TT = 3.31 ± 0.23	AA = 0.60 ± 0.21 AT = 0.73 ± 0.09 TT = 0.69 ± 0.08
LHA total^a^	*F*_(2, 919)_ = 0.18, *p* = 0.838, η^2^ <0.001	AT = 7.84 ± 0.82 AT = 8.28 ± 0.33 TT = 8.07 ± 0.29	*F*_(2, 916)_ = 0.86, *p* = 0.426, η^2^ = 0.002	AA = 10.50 ± 1.58 AT = 11.33 ± 0.60 TT = 10.34 ± 0.50	AA = 6.38 ± 0.75 AT = 6.11 ± 0.32 TT = 6.19 ± 0.29
**rs8062784**					
Aggression	*F*_(1, 920)_ = 1.19, *p* = 0.276, η^2^ = 0.001	AA = 5.95 ± 0.15 T-all = 6.43 ± 0.42	*F*_(2, 918)_ = 0.20, *p* = 0.654, η^2^ <0.001	AA = 7.02 ± 0.25 T-all = 7.61 ± 0.64	AA = 5.16 ± 0.18 T-all = 5.36 ± 0.52
Antisocial behavior	*F*_(1, 920)_ = 0.32, *p* = 0.574, η^2^ <0.001	AA = 1.89 ± 0.10 T-all = 2.06 ± 0.27	*F*_(2, 918)_ = 0.60, *p* = 0.439, η^2^ = 0.001	AA = 3.48 ± 0.18 T-all = 3.71 ± 0.48	AA = 0.72 ± 0.06 T-all = 0.55 ± 0.18
LHA total	*F*_(1, 920)_ = 0.86, *p* = 0.353, η^2^ = 0.001	AA = 8.07 ± 0.23 T-all = 8.68 ± 0.62	*F*_(2, 918)_ = 0.77, *p* = 0.382, η^2^ = 0.001	AA = 10.61 ± 0.40 T-all = 11.57 ± 1.04	AA = 6.18 ± 0.22 T-all = 6.05 ± 0.63
**rs12921846**					
Aggression	*F*_(2, 919)_ = 1.39, *p* = 0.249, η^2^ = 0.003	AA = 5.82 ± 0.18 AT = 6.27 ± 0.24 TT = 6.53 ± 0.68	*F*_(2, 916)_ = 0.14, *p* = 0.870, η^2^ <0.001	AA = 6.89 ± 0.29 AT = 7.41 ± 0.39 TT = 7.43 ± 1.23	AA = 5.03 ± 0.22 AT = 5.33 ± 0.30 TT = 6.04 ± 0.76
Antisocial Behavior	*F*_(2, 919)_ = 1.08, *p* = 0.341, η^2^ = 0.002	AA = 1.82 ± 0.12 AT = 2.10 ± 0.16 TT = 1.80 ± 0.45	*F*_(2, 916)_ = 1.55, *p* = 0.214, η^2^ = 0.003	AA = 3.30 ± 0.22 AT = 3.86 ± 0.29 TT = 3.64 ± 0.91	AA = 0.71 ± 0.07 AT = 0.66 ± 0.10 TT = 0.81 ± 0.26
LHA total	*F*_(2, 919)_ = 1.41, *p* = 0.246, η^2^ = 0.003	AA = 7.86 ± 0.27 AT = 8.57 ± 0.36 TT = 8.73 ± 1.02	*F*_(2, 916)_ = 0.65, *p* = 0.525, η^2^ = 0.001	AA = 10.31 ± 0.47 AT = 11.42 ± 0.62 TT = 11.14 ± 1.98	AA = 6.03 ± 0.26 AT = 6.24 ± 0.36 TT = 7.42 ± 0.93
**rs6500744**					
Aggression	*F*_(2, 919)_ = 1.89, *p* = 0.161, η^2^ = 0.004	CC = 5.94 ± 0.22 CT = 6.23 ± 0.21 TT = 5.41 ± 0.39	*F*_(2, 916)_ = 0.75, *p* = 0.474, η^2^ = 0.002	CC = 7.0 ± 0.36 CT = 7.51 ± 0.34 TT = 6.04 ± 0.62	CC = 5.11 ± 0.27 CT = 5.31 ± 0.25 TT = 4.90 ± 0.47
Antisocial behavior	*F*_(2, 919)_ = 0.22, *p* = 0.801, η^2^ <0.001	CC = 1.98 ± 0.15 CT = 1.88 ± 0.14 TT = 1.82 ± 0.25	*F*_(2, 916)_ = 1.22, *p* = 0.294, η^2^ = 0.003	CC = 3.52 ± 0.27 CT = 3.64 ± 0.26 TT = 3.07 ± 0.46	CC = 0.78 ± 0.09 CT = 0.60 ± 0.09 TT = 0.81 ± 0.16
LHA total	*F*_(2, 919)_ = 0.92, *p* = 0.400, η^2^ = 0.002	CC = 8.18 ± 0.33 CT = 8.31 ± 0.31 TT = 7.42 ± 0.58	*F*_(2, 916)_ = 1.18, *p* = 0.308, η^2^ = 0.003	CC = 10.67 ± 0.57 CT = 11.26 ± 0.55 TT = 9.20 ± 1.0	CC = 6.23 ± 0.33 CT = 6.17 ± 0.30 TT = 5.99 ± 0.58

a *The total score of LHA is greater than subscales aggression and antisocial behavior together because it also contains self-directed aggressiveness. The latter scale is strongly skewed toward non-occurrence of the behavior and did not reveal any significant genotype effect by non-parametric tests*.

### Personality and *RBFOX1* Genotype

Neuroticism was associated with the *RBFOX1* rs8062784, being lower in homozygotes for the aggressiveness risk allele A (Table 4). *RBFOX1* rs809682 was associated with extraversion, while the homozygotes for the risk allele T having higher scores. No other polymorphism was statistically significantly associated with neuroticism or extraversion.

**Table 4:** The effects of *RBFOX1* genotypes on neuroticism and extraversion at age 25.

	**Genotype main effects**	
	**Main statistics**	**Scores**
**rs809682**		
Neuroticism	*F*_(2, 853)_ = 0.57, *p* = 0.565, η^2^ = 0.001	AA = 73.5 ± 3.5 AT = 75.1 ± 1.4 TT = 73.3 ± 1.3
Extraversion	***F***_**(2, 853)**_ **=** **5.0,** ***p*** **=** **0.007,** **η**^2^ **=** **0.012**	AA = 105.6 ± 3.1 AT = 113.8 ± 1.3 TT = 115.7 ± 1.1
**rs8062784**		
Neuroticism	***F***_**(1, 854)**_ **=** **7.20,** ***p*** **=** **0.007,** **η**^2^ **=** **0.008**	AA = 73.1 ± 1.0 T-all = 81.0 ± 2.8
Extraversion	*F*_(1, 854)_ = 1.98, *p* = 0.160, η^2^ = 0.002	AA = 114.6 ± 0.8 T-all = 111.0 ± 2.5

*Bold denotes statistically significant difference between groups*.

### *RBFOX1* Polymorphisms and the Occurrence of Alcohol Use Disorder

**Figure 1:**
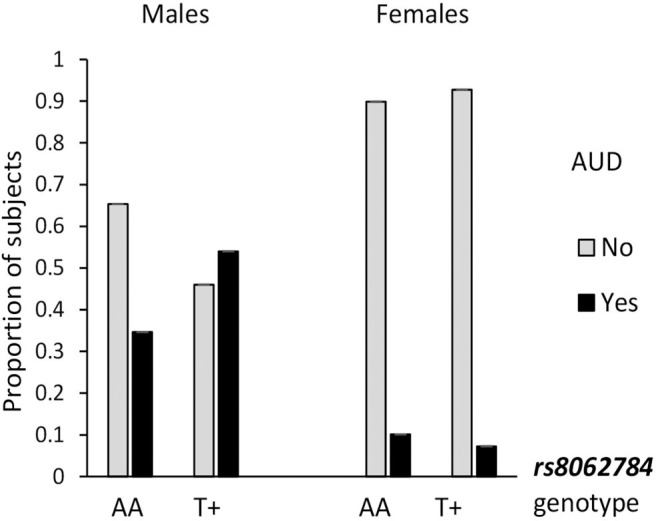
Association of *RBFOX1 rs8062784* with alcohol use disorder in males. Males, χ^2^ = 7.01; *p* = 0.008 (*n* = 411); females, χ^2^ = 0.45; *p* = 0.509 (*n* = 520). Lifetime diagnosis is based on MINI interview at age 25. AUD in 158 males out of 413 and 54 females out of 523 (in total, 212 out of 936).

**Figure 2:**
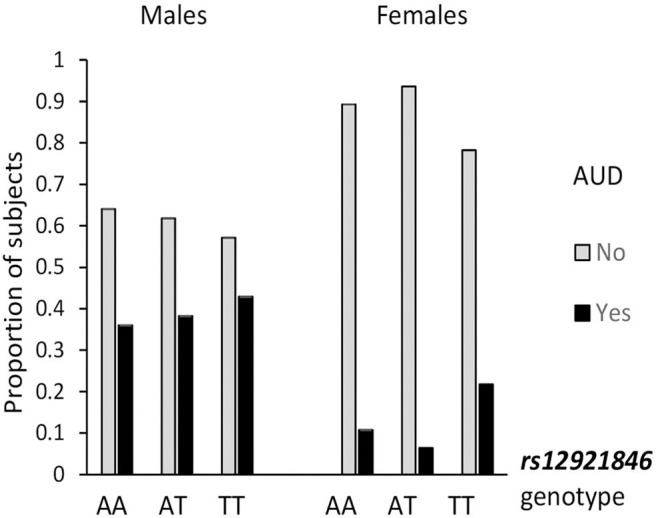
Association of *RBFOX1 rs12921846* with alcohol use disorder in females. Males, χ^2^ = 0.41; *p* = 0.815 (*n* = 411); females, χ^2^ = 6.22; *p* = 0.045 (*n* = 520). Lifetime diagnosis is based on MINI interview at age 25. AUD in 158 males out of 413 and 54 females out of 523 (in total, 212 out of 936).

Alcohol use is a most salient mediator to aggressiveness, so the association of *RBFOX1* polymorphisms with lifetime prevalence of alcohol use disorder by age 25 was examined. The overall genotype effect (χ^2^ = 4.14; *p* = 0.042) was revealed for *RBFOX1* rs8062784 (Figure 1): This was largely based on male subjects carrying the less frequent low aggressiveness allele T who had almost twice higher risk of alcohol abuse. While analyzing males and females separately, we found that rs12921846 (Figure 2) was associated with alcohol use disorder in females (χ^2^ = 4.22; *p* = 0.045). Female *RBFOX1* rs12921846 homozygotes for the less frequent T-allele, also has been related to lower aggressiveness, had higher alcohol abuse risk.

### Alcohol Use Disorder, Sociodemographic Factors, Personality, and the *RBFOX1* rs8062784 Genotype

Next we examined the association of several factors potentially related to alcohol use disorder at age 25, which is the last observation for both cohorts. AUD was more prevalent in males: 10% of women and 38% of men had experienced AUD by age 25 (χ^2^ = 102.8; df = 1; *p* < 0.001). In these birth cohorts, lifetime alcohol use disorder was not associated with income in either male or female subjects (data not shown), probably owing to early age. At age 25, 58% of the participants were living with a spouse, but there was no difference in lifetime occurrence of AUD between subjects who lived with their spouse (21%) compared with subjects who did not (25%). However, the lifetime prevalence of AUD was higher (15%; n = 29) in females who did not live with a spouse compared with those who did (8%; χ^2^ = 6.3; df = 1; *p* = 0.01). There was a higher prevalence of AUD among subjects with lower education level at age 25 (32 vs. 17%; χ^2^ = 28.99; df = 1; *p* < 0.001; males, 48 vs. 30%; χ^2^ = 15.35; df = 1; *p* < 0.001; females, 14 vs. 9%; χ^2^ = 3.85; df = 1; *p* = 0.050); but *RBFOX1* genotype was not associated with education (data not shown).

**Figure 3:**
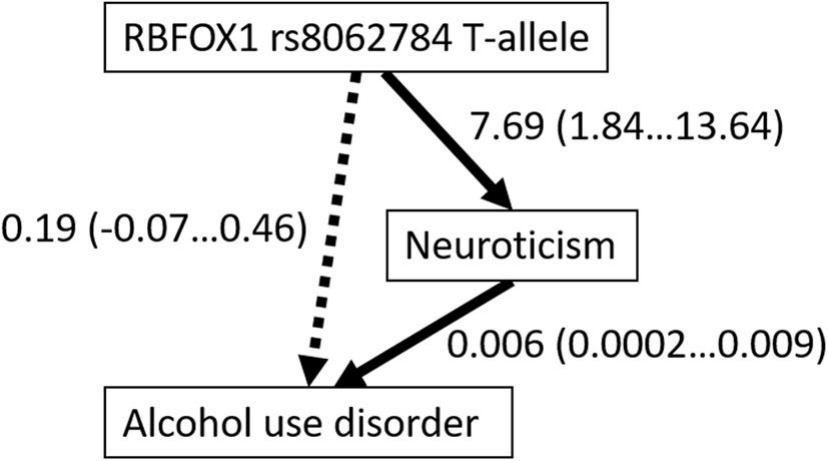
Mediation by neuroticism of the association of *RBFOX1 rs8062784* and alcohol use disorder. Regression weights with credible intervals (95%) are shown.

No interaction between the gender factor and lifetime alcohol use disorder was found for the Big Five personality traits (Table 5), but participants with AUD had significantly higher neuroticism [F_(1, 781)_ = 30.01; *p* < 0.001; η^2^ = 0.037], lower agreeableness [F_(1, 774)_ = 9.39; *p* = 0.002; η^2^ = 0.012], and lower conscientiousness [F_(1, 779)_ = 18.15; *p* < 0.001; η^2^ = 0.023]. Because the *RBFOX1* rs8062784 genotype and occurrence of alcohol use disorder were both associated with neuroticism, we examined whether or not the association of the *RBFOX1* genotype could be mediated by this personality trait. According to a Bayesian model by the Markov chain Monte Carlo method, the *RBFOX1* rs8062784 direct effect on AUD was not significant (regression weight 0.19; Bayes' credible interval −0.069…0.455; probability 95%), the *RBFOX1* rs8062784 effect on Neuroticism was 7.69 (Bayes' credible interval 1.84…13.64; probability 95%), and the neuroticism effect on AUD was 0.006 (0.0002…0.009), suggestive of the neuroticism mediated association of *RBFOX1* with alcohol use disorder (Figure 3).

**Table 5:** Personality traits at age 25 by gender and lifetime alcohol use disorder (AUD).

	**Females, no AUD**	**Females with AUD**	**Males, no AUD**	**Males with AUD**
Neuroticism	75.3 ± 1.3	92.1 ± 4.1	66.1 ± 1.6	76.1 ± 2.3
Extraversion	115.3 ± 1.2	118.1 ± 2.8	111.5 ± 1.6	115.7 ± 2.0
Openness	126.8 ± 1.0	127.9 ± 2.4	117.0 ± 1.3	115.7 ± 1.7
Agreeableness	129.5 ± 0.9	121.2 ± 2.6	118.3 ± 1.3	116.3 ± 1.6
Conscientiousness	130.4 ± 1.1	119.0 ± 3.4	124.8 ± 1.5	119.2 ± 1.7

## Discussion

Four polymorphisms of the *RBFOX1* gene, previously linked to aspects of aggressive behavior, were in the ECPBHS sample not associated with aggressiveness in young adulthood, neither by self-report nor interview measurement. Genome-wide association studies indicate a very small effect of each gene on aggressiveness in human population, so given the size of the sample, this may not be surprising. Nevertheless, when performing subgroup analyses by ethnicity, age of participants, sample characteristics, and outcome measures, significant associations emerge (6). Several variants in a number of candidate genes have been found strongly associated with aggression measures in the ECPBHS sample [see (29, 33) and references therein]. It should hence be concluded that in this specific population of which the ECPBHS sample is highly representative, other genes than *RBFOX1* play a role in aggressiveness. Thus, other genetic variants than the ones inspected play a role in aggressiveness in this sample, although we cannot exclude other variants in *RBFOX1* not investigated here.

Neuroimaging genetic studies support the notion that the *RBFOX1* gene contributes to brain function and structure. The expression levels of *RBFOX1* in the brain are highest in the anterior cingulate cortex that regulates emotions and social behavior (38–41). The thickness of the ACC has been associated with aggressiveness (41, 42), and in individuals prone to aggression, its activity in response to provocation is attenuated (43). Furthermore, higher activity of ACC has been found in aggressive adolescents with disruptive behavior disorders (44). Yet anterior cingulate is involved in the whole complexity of emotion regulation, and the meaning of any alteration at the molecular level likely depends on the overall state of the whole complex [e.g., (45)]. Because previous studies that have implicated these specific variants of *RBFOX1* were either on selected groups of participants or relied on specific measures of phenotype, we took the approach to study the association of *RBFOX1* with basic personality traits that could provide insight into which factors play a role in the potential effect of *RBFOX1*. Two of the polymorphisms were nominally associated with such basic personality traits as neuroticism and extraversion.

Neuroticism was lower with two A-alleles of *RBFOX1* rs8062784. Previously, this allele was associated with anger in a GWAS that was targeted at hostility (25). Anger or hostility is indeed a component of neuroticism in the five-factor model. For this reason, we separately analyzed the six facets of neuroticism and found four of them, including anger/hostility significantly associated with the genotype (data not shown). Interestingly, the weakest of the subscale associations was with impulsivity, often a facilitative factor in aggressive behavior. While this association of genotype and neuroticism could well-be a mere chance finding, its direction also supports the hypothesis that low neuroticism in the risk genotype in the present sample is a mechanism preventing overt aggressiveness: In population-derived samples, aggressive subjects appear to have higher neuroticism (46–48).

*RBFOX1* rs809682 was associated with extraversion: Homozygotes for the T-allele had higher scores. The meta-analysis of Pappa et al. (7) of nine population-based GWASs of 19,000 children suggested the major T allele to carry the aggression risk. Aggressiveness rating in these studies was based on maternal scores, and this may differ from self-assessment and interviews; moreover, levels of continuity of aggression from childhood to early adulthood have been found to be only moderate (49). Furthermore, in a study of adult aggressive prisoners, a significant association was found for rs809682; however, the direction of the effect was opposite as in another (7) study, this time with the minor A allele linked to aggression (8), although the sample size was very limited. These conflicting results may also find an explanation in the dual potential of extraversion to moderate aggressiveness. Extraversion is the tendency toward interaction with others, assertiveness, liveliness, and action-orientation (50, 51). While aspects of extraversion are defined as deriving from positive emotionality, another central part of extraversion is assertiveness. Frost (52) made the early notion that extraverts tend to be more assertive and lacking in submissiveness and self-criticism. Assertiveness and verbal aggression have been demonstrated to correlate positively (53), and extraversion has recently been shown to be positively associated with physical aggression (54). Indeed, in our exploratory analysis of the facets of extraversion, the *RBFOX1* rs809682 T/T homozygocity was related rather to assertiveness, activity, gregariousness, and positive emotions facets but less to friendliness and excitement seeking (data not shown). Thus, somewhat speculatively, lower prevalence of rs809682 T-allele among less aggressive subjects in some studies may indirectly suggest that this allele is promoting assertive behavior, and the absence of it mitigates aggressive behavioral choices.

Two of the *RBFOX1* variants were associated with alcohol use disorder, rs8062784 in males and rs12921846 in females. In both occasions, higher prevalence of alcohol use disorder was present with the minor variant previously associated with lower level aggression. Problematic alcohol use is often predictive of violent behavior, but in this sample, the risk alleles were not associated with aggressiveness. This result rather converges with the personality findings in that in the risk allele carriers, who constitute large majority of subjects, aggressiveness is not common owing to the generally positive side of these variants. According to this scenario, the *RBFOX1* aggressiveness-related variants owe this association to other coinciding genetic or environmental factors. In the present sample, alcohol use disorder was associated with both rs8062784 genotype and neuroticism, and the genotype also with neuroticism. A path analysis supported the possibility of mediation by neuroticism between the genotype and alcohol use disorder. Speculatively, the major rs8062784 allele, promoting lower neuroticism and protective of alcohol use disorder in this sample, may in general be associated with proactive behaviors and thus become associated with anger in different types of environment where the positive side of agonistic behavior cannot be properly channeled.

Analysis simultaneously involving four gene polymorphisms and many comparisons inflates the possibility of false positive findings, and conventional correction for multiple testing would render all associations non-significant. This is a major limitation of the present longitudinal study, in that it is restricted in its sample size. *RBFOX1* has previously been associated with aggression-related phenotypes in a number of GWAS. The present sample is much smaller, but nevertheless, any large direct effect of the genotype would have been detected. We do, however, suggest a few potential mediating mechanisms by which the variants of the *RBFOX1* gene may exert an indirect and therefore small effect on aggression that becomes revealed in large samples. The strengths of the study are its standard of data collection performed in the uniform conditions of a laboratory, the strong representation of the regional population, and the solid rationale behind the selection of *RBFOX1* as a target. Thus, the findings that *RBFOX1* variants appear to be associated with personality traits and alcohol use disorder merit attention in further studies.

## Data Availability Statement

The datasets generated for this study are available on request to the corresponding author.

## Ethics Statement

The studies involving human participants were reviewed and approved by Ethics Review Committee on Human Research of the University of Tartu. The patients/participants provided their written informed consent to participate in this study.

## Author Contributions

NF-C, SF, BF, AR, BC, and JH contributed to the conception and design of the study. KL, TK, and JH selected and prepared the questionnaires, and KL conducted interviews. MV, KL, TK, MK, TV, and JH collected the data. MV, KL, and L-MT analyzed the data. MV performed genotyping. MV and JH wrote the manuscript. All authors were involved in the final editing and have approved the manuscript.

## Conflict of Interest

The authors declare that the research was conducted in the absence of any commercial or financial relationships that could be construed as a potential conflict of interest.

## References

[1] UN World Health Organization Global Status Report on Violence Prevention. (2014). Available online at: http://www.refworld.org/docid/54aa8de14.html (accessed March 11, 2018).

[2] Georgiev AV Klimczuk ACE Traficonte DM Maestripieri D When violence pays: a cost-benefit analysis of aggressive behavior in animals and humans. Evol Psychol. (2013) 11:678–99. 10.1177/147470491301100313 23864299 PMC3859192

[3] Gómez JM Verdú M González-Megías A Méndez M. The phylogenetic roots of human lethal violence. Nature. (2016) 538:233–7. 10.1038/nature19758 27680701

[4] Veroude K Zhang-James Y Fernàndez-Castillo N Bakker MJ Cormand B Faraone SV. Genetics of aggressive behavior: an overview. Am J Med Genet B Neuropsychiatr Genet. (2016) 171B:3–43. 10.1002/ajmg.b.32364 26345359

[5] Asherson P Cormand B. The genetics of aggression: where are we now? Am J Med Genet B. (2016) 171B:559–61. 10.1002/ajmg.b.32450 27061441

[6] Vassos E Collier DA Fazel S. Systematic meta-analyses and field synopsis of genetic association studies of violence and aggression. Mol Psychiatry. (2014) 19:471–7. 10.1038/mp.2013.31 23546171 PMC3965568

[7] Pappa I St Pourcain B Benke K Cavadino A Hakulinen C Nivard MG. A genome-wide approach to children's aggressive behavior: the EAGLE consortium. Am J Med Genet Part B Neuropsychiatr Genet. (2016) 171:562–72. 10.1002/ajmg.b.32333 26087016

[8] Fernàndez-Castillo N Cormand B. Aggressive behavior in humans: Genes and pathways identified through association studies. Am J Med Genet B Neuropsychiatr Genet. (2016) 171:676–96. 10.1002/ajmg.b.32419 26773414

[9] Davies MN Verdi S Burri A Trzaskowski M Lee M Hettema JM. Generalised anxiety disorder – a twin study of genetic architecture, genome-wide association and differential gene expression. PLoS ONE. (2015) 10:e0134865. 10.1371/journal.pone.0134865 26274327 PMC4537268

[10] Fogel BL Wexler E Wahnich A Friedrich T Vijayendran C Gao F. RBFOX1 regulates both splicing and transcriptional networks in human neuronal development. Hum Mol Genet. (2012) 21:4171–86. 10.1093/hmg/dds240 22730494 PMC3441119

[11] Lukong KE Chang KW Khandjian EW Richard S. RNA-binding proteins in human genetic disease. Trends Genet. (2008) 24:416–25. 10.1016/j.tig.2008.05.004 18597886

[12] Glisovic T Bachorik JL Yong J Dreyfuss G. RNA-binding proteins and post-transcriptional gene regulation. FEBS Lett. (2008) 582:1977–86. 10.1016/j.febslet.2008.03.004 18342629 PMC2858862

[13] Jin Y Suzuki H Maegawa S Endo H Sugano S Hashimoto K. A vertebrate RNA-binding protein Fox-1 regulates tissue-specific splicing via the pentanucleotide GCAUG. EMBO J. (2003) 22:905–12. 10.1093/emboj/cdg089 12574126 PMC145449

[14] Anantharaman V Koonin EV Aravind L. Comparative genomics and evolution of proteins involved in RNA metabolism. Nucleic Acids Res. (2002) 30:1427–64. 10.1093/nar/30.7.1427 11917006 PMC101826

[15] Darnell JC Richter JD. Cytoplasmic RNA-binding proteins and the control of complex brain function. Cold Spring Harb Perspect Biol. (2012) 4:a012344. 10.1101/cshperspect.a012344 22723494 PMC3405866

[16] Lee JA Damianov A Lin CH Fontes M Parikshak NN Anderson ES. Cytoplasmic Rbfox1 regulates the expression of synaptic and autism-related genes. Neuron. (2015) 89:113–28. 10.1016/j.neuron.2015.11.025 26687839 PMC4858412

[17] Hamada N Ito H Nishijo T Iwamoto I Morishita R Tabata H. Essential role of the nuclear isoform of RBFOX1, a candidate gene for autism spectrum disorders, in the brain development. Sci Rep. (2016) 6:30805. 10.1038/srep30805 27481563 PMC4969621

[18] Elia J Gai X Xie HM Perin JC Geiger E Glessner JT. Rare structural variants found in attention-deficit hyperactivity disorder are preferentially associated with neurodevelopmental genes. Mol Psychiatry. (2010) 15:637–46. 10.1038/mp.2009.57 19546859 PMC2877197

[19] Hamshere ML Green EK Jones IR Jones L Moskvina V Kirov G. Genetic utility of broadly defined bipolar schizoaffective disorder as a diagnostic concept. Br J Psychiatry. (2009) 195:23–9. 10.1192/bjp.bp.108.061424 19567891 PMC2802523

[20] Xu B Roos JL Levy S van Rensburg EJ Gogos JA Karayiorgou M. Strong association of *de novo* copy number mutations with sporadic schizophrenia. Nat Genet. (2008) 40:880–5. 10.1038/ng.162 18511947

[21] Bhalla K Phillips HA Crawford J McKenzie OL Mulley JC Eyre H. The *de novo* chromosome 16 translocations of two patients with abnormal phenotypes (mental retardation and epilepsy) disrupt the A2BP1 gene. J Hum Genet. (2004) 49:308–11. 10.1007/s10038-004-0145-4 15148587

[22] Vounou M Janousova E Wolz R Stein JL Thompson PM Rueckert D. Sparse reduced-rank regression detects genetic associations with voxel-wise longitudinal phenotypes in Alzheimer's disease. Neuroimage. (2012) 60:700–16. 10.1016/j.neuroimage.2011.12.029 22209813 PMC3551466

[23] Fernàndez-Castillo N Gan G van Donkelaar MMJ Vaht M Weber H Retz W. RBFOX1, encoding a splicing regulator, is a candidate gene for aggressive behavior. Eur Neuropsychopharmacol. (2020) 30:44–55. 10.1016/j.euroneuro.2017.11.012 29174947 PMC10975801

[24] Sonuga-Barke EJS Lasky-Su J Neale BM Oades R Chen W Franke B. Does parental expressed emotion moderate genetic effects in ADHD? an exploration using a genome wide association scan. Am J Med Genet B Neuropsychiatr Genet. (2008) 147B:1359–68. 10.1002/ajmg.b.30860 18846501

[25] Merjonen P Keltikangas-Järvinen L Jokela M Seppälä I Lyytikäinen LP Pulkki-Råback L. Hostility in adolescents and adults: a genome-wide association study of the Young Finns. Transl Psychiatry. (2011) 1:e11. 10.1038/tp.2011.13 22832427 PMC3309462

[26] Anney RJL Lasky-Su J Ó'Dúshláine C Kenny E Neale BM Mulligan A. Conduct disorder and ADHD: evaluation of conduct problems as a categorical and quantitative trait in the international multicenter ADHD genetics study. Am J Med Genet B Neuropsychiatr Genet. (2008) 147:1369–78. 10.1002/ajmg.b.30871 18951430

[27] Young R Sweeting H West P. A longitudinal study of alcohol use and antisocial behaviour in young people. Alcohol Alcohol. (2008) 43:204–14. 10.1093/alcalc/agm147 17977868 PMC2367698

[28] Harro M Eensoo D Kiive E Merenäkk L Alep J Oreland L. Platelet monoamine oxidase in healthy 9- and 15-years old children: the effectof gender, smoking and puberty. Prog Neuropsychopharmacol Biol Psychiatry. (2001) 25:1497–511. 10.1016/S0278-5846(01)00212-3 11642650

[29] Harro J Laas D Eensoo D Kurrikoff T Sakala K Vaht M. Orexin/hypocretin receptor gene (*HCRTR1*) variation is associated with aggressive behaviour. Neuropharmacology. (2019) 156:107527. 10.1016/j.neuropharm.2019.02.009 30742846

[30] Laas K Kiive E Mäestu J Vaht M Veidebaum T Harro J. Nice guys: homozygocity for the TPH2−703G/T (rs4570625) minor allele promotes low aggressiveness and low anxiety. J Affect Disord. (2017) 215:230–6. 10.1016/j.jad.2017.03.045 28342337

[31] Buss AH Perry M. The aggression questionnaire. J Pers Soc Psychol. (1992) 63:259–452. 10.1037/0022-3514.63.3.452 1403624

[32] Coccaro EF Berman ME Kavoussi RJ. Assessment of life history of aggression: development and psychometric characteristics. Psychiatry Res. (1997) 73:147–57. 10.1016/S0165-1781(97)00119-4 9481806

[33] O'Leary A Laas K Vaht M Kiive M Veidebaum T Reif A. Nitric oxide synthase genotype interacts with stressful life events to increase aggression in male subjects in a population-representative sample. Eur Neuropsychopharmacol. (2020) 30:56–65. 10.1016/j.euroneuro.2019.07.241 31405541

[34] Costa PT Jr McCrae RR The NEO-PI/NEO-FFI Manual Supplement. Odessa, FL: Psychological Assessment Resources (1989).

[35] Mõttus R Pullmann H Allik J Toward more readable big five personality inventories. Eur J Psychol Assess. (2006) 22:149–57. 10.1027/1015-5759.22.3.149

[36] Sheehan DV Lecrubier Y Sheehan KH Amorim P Janavs J Weiller E. The Mini-International Neuropsychiatric Interview (MINI): the development and validation of a structured diagnostic psychiatric interview for DSM-IV and ICD-10. J Clin Psychiatry. (1998) 59(Suppl. 20):22–33. 9881538

[37] Shlik J Aluoja A Kihl E MINI 5.0.0. Mini Rahvusvaheline Neuropsühhiaatriline Intervjuu DSM –IV. Estonian version of MINI international neuropsychiatric interview. Tartu (1999).

[38] Botvinick MM. Conflict monitoring and decision making: reconciling two perspectives on anterior cingulate function. Cogn Affect Behav Neurosci. (2007) 7:356–66. 10.3758/CABN.7.4.356 18189009

[39] Devinsky O Morrell MJ Vogt BA. Contributions of anterior cingulate cortex to behaviour. Brain. (1995) 118 (Pt. 1):279–306. 10.1093/brain/118.1.279 7895011

[40] Carretié L Albert J López-Martín S Tapia M. Negative brain: an integrative review on the neural processes activated by unpleasant stimuli. Int J Psychophysiol. (2009) 71:57–63. 10.1016/j.ijpsycho.2008.07.006 18727941

[41] Yang Y Joshi SH Jahanshad N Thompson PM Baker LA. Neural correlates of proactive and reactive aggression in adolescent twins. Aggress Behav. (2017) 43:230–40. 10.1002/ab.21683 27766650 PMC6192547

[42] Ducharme S Hudziak JJ Botteron KN Ganjavi H Lepage C Collins DL. Right anterior cingulate cortical thickness and bilateral striatal volume correlate with child behavior checklist aggressive behavior scores in healthy children. Biol Psychiatry. (2011) 70:283–90. 10.1016/j.biopsych.2011.03.015 21531391 PMC3134605

[43] Bufkin JL Luttrell VR. Neuroimaging studies of aggressive and violent behavior: current fndings and implications for criminology and criminal justice. Trauma Violence Abuse. (2005) 6:176–91. 10.1177/1524838005275089 15753199

[44] Cohn MD Popma A van den Brink W Pape LE Kindt M van Domburgh L. Fear conditioning, persistence of disruptive behavior and psychopathic traits: an fMRI study. Transl Psychiatry. (2013) 3:e319. 10.1038/tp.2013.89 24169638 PMC3818535

[45] Harro J Marcusson J Oreland L. Alterations in brain cholecystokinin receptors in suicide victims. Eur Neuropsychopharmacol. (1992) 2:57–63. 10.1016/0924-977X(92)90037-9 1638175

[46] Caprara GV Barbaranelli C Zimbardo P Understanding the complexity of human aggression: Affective, cognitive and social dimensions of individual differences in propensity toward aggression. Eur J Pers. (1996) 10:133–55. 10.1002/(SICI)1099-0984(199606)10:2<133::AID-PER252>3.0.CO;2-E

[47] Tremblay PF Ewart LA the buss and perry aggression questionnaire and its relations to values, the Big Five, provoking hypothetical situations, alcohol consumption patterns, and alcohol expectancies. Pers Indiv Diff. (2005) 38:337–48. 10.1016/j.paid.2004.04.012

[48] KodŽopeljić J Smederevac S Mitrović D Dinić B Colović P. School bullying in adolescence and personality traits: a person-centered approach. J Interpers Violence. (2014) 29:736–57. 10.1177/0886260513505216 24255068

[49] Huesmann LR Dubow EF Boxer P. Continuity of aggression from childhood to early adulthood as a predictor of life outcomes: implications for the adolescent-limited and life-course-persistent models. Aggress Behav. (2009) 35:136–49. 10.1002/ab.20300 19189380 PMC4513937

[50] McCrae RR Costa PT Handbook of personality: Theory and research. 2nd Edn New York, NY: Guilford Press (1999).

[51] McCrae RR John OP. An introduction to the five-factor model and its applications. J Pers. (1992) 60:175–215. 10.1111/j.1467-6494.1992.tb00970.x 1635039

[52] Frost BP. On the relationship between extraversion and aggression. Psychol Rep. (1981) 49:1009–10. 10.2466/pr0.1981.49.3.1009 7330138

[53] Galassi JP Galassi MD. Relationship between assertiveness and aggressiveness. Psychol Rep. (1975) 36:352–4. 10.2466/pr0.1975.36.2.352 1144578

[54] Cavalcanti JG Pimentel CE Personality and aggression: a contribution of the general aggression model. Estud Psicol. (2016) 33:443–51. 10.1590/1982-02752016000300008

